# Drilling into the functional significance of stereopsis: the impact of stereoscopic information on surgical performance

**DOI:** 10.1111/opo.12393

**Published:** 2017-06-27

**Authors:** Loulwa M. Al‐Saud, Faisal Mushtaq, Isra'a Mirghani, Ahmed Balkhoyor, Andrew Keeling, Michael Manogue, Mark A. Mon‐Williams

**Affiliations:** ^1^ School of Psychology Faculty of Medicine & Health University of Leeds Leeds UK; ^2^ School of Dentistry Faculty of Medicine & Health University of Leeds Leeds UK; ^3^ College of Dentistry King Saud University Riyadh Saudi Arabia; ^4^ Bradford Institute of Health Research Bradford UK; ^5^ Norwegian Centre for Vision University of Southeast Norway Kongsberg Norway

**Keywords:** motor performance, stereo, stereopsis, virtual reality simulators

## Abstract

**Purpose:**

One suggested advantage of human binocular vision is the facilitation of sophisticated motor control behaviours via stereopsis – but little empirical evidence exists to support this suggestion. We examined the functional significance of stereopsis by exploring whether stereopsis is used to perform a highly skilled real‐world motor task essential for the occupational practice of dentistry.

**Methods:**

We used a high fidelity virtual reality simulator to study how dentists’ performance is affected by the removal of horizontal retinal image disparities under direct and indirect (mirror) observation. Thirteen qualified dentists performed a total of four different dental tasks under non‐stereoscopic and stereoscopic vision conditions, with two levels of task complexity (direct and indirect observation) using a virtual reality dental simulator.

**Results:**

Depth related errors were significantly higher under non‐stereoscopic viewing but lateral errors did not differ between conditions. Indirect observation led to participants drilling less of the target area compared to direct viewing, but this did not interact with the stereopsis manipulation.

**Conclusions:**

The data confirm that dental practitioners use stereopsis and its presence results in improved dental performance. It remains to be determined whether individuals with stereo‐deficits can compensate adequately. Nevertheless, these findings suggest an important role for stereopsis within at least one occupation and justify the design of simulators with 3D displays.

## Introduction

What is the functional significance of stereopsis? Horizontal retinal image disparities can provide information on the depth, slant and curvature of objects.^1^ This information is known to be phenomenology salient, but the more critical issue is whether such information supports activities of daily living. The issue is important from a clinical perspective as uncorrected refractive error, strabismus and amblyopia have the potential to reduce stereoacuity thresholds. A debate on the functional advantages of binocular stereopsis has persisted for decades,^2^ and evidence of its importance has accumulated.^3^ There remains a notable absence of studies, however, where the role of fine stereopsis is related to an individual's ability to carry out a professional task in naturalistic settings (i.e. where other information is available). In the context of medical advances, this is a remarkable state of affairs as it means that the ‘costs’ of treating conditions such as strabismus and amblyopia are difficult to model from a health economic perspective.

One major difficulty with investigating the role of stereopsis in real‐world tasks is that binocular viewing confers a number of advantages. For example, a binocular view provides vergence information that can be used to gauge the egocentric distance of a fixated target.^4^ Two eyes also enable a wider field of view and improved perceptual thresholds (the square root of two advantage). This means that covering one eye to remove binocular vision does not provide an appropriate experimental manipulation for the purpose of establishing the contribution of stereopsis to a given task. Moreover, this approach increases the difficulty in interpreting any decrement in performance following this manipulation.^5^ There are, however, some useful alternative approaches to investigating the role of binocular viewing: (i) selectively interfering with binocular cues to target distance (e.g. vergence with prisms^6^); (ii) selectively interfering with binocular cues to depth (e.g. spatially degraded images^7^); (iii) selectively interfering with binocular cues to distance and depth (i.e. bi‐ocular viewing^4^, ^8^); (iv) comparing performance of individuals with and without stereo‐deficits on tasks involving manual dexterity^9^, ^10^, ^11^; (v) using Virtual Reality systems to manipulate binocular cues whilst leaving other information unaffected.^12^ The overall conclusion that emerges from studies employing these techniques is that, generally, but not always, stereopsis provides useful information that supports skilled performance in certain visuomotor tasks. For example, it has been found that reach‐to‐grasp movements are slower and less accurate under biocular viewing, with biocular viewing showing similar degradation to monocular viewing relative to a normal binocular view.^13^

Such findings raise the issue of whether degraded performance on experimental tasks has any significance in terms of activity limitation or participation restriction (given the WHO framework on disability identifies activity limitation and participation restriction as key features of disability). One particularly germane aspect of this issue is whether stereopsis is required for specific professional activities. Findings from some studies did not support the need for high‐grade stereopsis in practicing surgeons^14^ or in simulated video‐assisted (2D) surgery.^15^ More recently, there has been a debate within the dental literature on whether adequate stereoacuity sensitivity should be a pre‐requisite for admission to dental training.^16^, ^17^ Unfortunately, there are no studies that have been able to provide empirical data to resolve this debate.^17^

It seems reasonable to suppose that stereopsis might be important to the practising dentist, since dentistry often involves working in a limited small‐scale visual environment and performing fine manipulations that require a high degree of hand‐eye coordination (working on a scale of tenths of a millimetre). Good depth perception appears to be particularly important because it enables the dentist to judge precisely the position of handheld objects, estimate relative distances and gauge correct sizes during dental performance.^18^ Moreover, good depth perception, intuitively at least, is an important asset in: the clinical diagnosis of dental caries, the estimation of correct convergence in crown preparation, oral radiography interpretation and in controlling various cavity preparations.^19^, ^20^, ^21^, ^22^

The intuition that stereopsis might be important in dentistry is reflected in the design of dental surgical simulators which provide realistic haptic feedback and 3D rendered images for the purpose of training. There is no doubt that the provision of 3D stereovision provides a powerful and realistic rendition of the oral cavity.^23^ Nevertheless, there are no data to support the inclusion of such perceptual information within the design of these surgical simulators (i.e. there is no evidence to demonstrate that dentists actually use stereo information to carry out surgical procedures – as previously discussed). The existence of such simulators does, however, provide a unique opportunity to address the fundamental issue of whether stereopsis has a functional role within an occupational activity. The Moog ‘Simodont’ virtual reality simulator presents an opportunity to examine this question as it can be engineered to provide a full binocular experience with (‘binocular’) or without (‘bi‐ocular’) stereoscopic viewing. This enables a robust investigation into the impact of removing stereoscopic information whilst keeping the other visual features of the display constant. It is worth noting that an earlier study attempted to address the issue of the functional significance of stereopsis in dental students.^23^ First year dental students were reported to show superior performance in binocular viewing relative to bi‐ocular in manual dexterity exercises undertaken using the Simodont. This finding seems to support a functional role for stereopsis in dentistry. Unfortunately, however, the design of the study precludes such a conclusion as there was an experimental confound introduced by half of the students being trained under binocular conditions whilst the other half were trained under bi‐ocular conditions. It is also difficult to interpret the findings from first year dental students (who are essentially untrained) as the data might reflect the learning process rather than the learned behaviour. There are also existing studies that show an impact of removing a binocular view within laparoscopic training devices^24^, ^25^ although studies to date have not used actual surgical procedures with fully trained surgeons. These findings are highly suggestive, but there is an urgent need to establish empirically whether trained professionals rely on stereopsis within a canonical professional procedure.

We therefore aimed to measure the impact of removing stereopsis within the display (whilst leaving other information unaffected) on the performance of qualified dentists in a standard dental task (drilling to prepare a cavity). We hypothesised that the removal of stereopsis should cause increased drilling errors in depth but not impact on the lateral errors if stereopsis has a functional role in this fundamental dental skill. We also investigated performance under direct observation and when the dentists needed to view the preparation within a mirror (a standard task requirement for dental practitioners).

## Methods

### Participants

Sixteen participants (13 female and 3 male, mean age = 32.4 years, S.D. = 3.3 years), with at least three years of clinical experience, but no previous experience of using a dental VR simulator, participated voluntarily in the study. Two participants were left handed (which is as easily accommodated within the simulator as in real‐world dentistry). All participants provided informed consent and were fully debriefed. The study followed the tenets of the Declaration of Helsinki and was approved by the local Research Ethics Committee at the School of Dentistry, University of Leeds.

### Stereoacuity screening

We wanted to ensure that our participants had access to stereo‐information before exploring whether removal of such information has an impact on real‐world performance. We therefore measured stereoacuity on a tablet (iPad 2, Model number A1395) running StereoTAB v3.0.4 (http://www.test-eye.com/index.php/stereotab).^26^ StereoTAB is a digital automated random‐dot based stereo test specifically designed for measuring global stereopsis (in seconds of arc). This non‐invasive test requires anaglyph eyeglasses and displays stereo‐images embedded in a background of random dots. This test has shown to have discriminative power and correlates well with the TNO stereo test.^27^ The tablet was placed 1 metre in front of the participant in a bespoke housing unit (so that distance and lighting conditions were standardised). A descending staircase procedure was used, whereby the stereoacuity level gradually decreased with each new stimulus. The minimum binocular disparity we could measure was 40″. Sixteen participants had no history of ophthalmological or neurological disorder and the use of the StereoTAB allowed us to ensure that the stereoacuity levels were within the normal range (the measured range in our participants was 40″–79″). Three participants scored outside of this range and their data were removed from analysis‐ leaving 13 participants for data analysis. It is quite possible that these participants had reasonable stereoacuity and the poor performance related to a difficulty with the test (the participants had no history of ophthalmic problems). Nevertheless, we considered it important to ensure that all participants included in our experiment had the ability to detect stereo‐information provided through computer generated images.

### Simodont VR haptic dental simulator

Participants’ motor performance was tested on the Simodont VR haptic dental simulator (The MOOG Industrial Group; www.moog.com). The simulator provides haptic force feedback based on the admittance control paradigm of the HapticMaster^28^ and records real‐time kinematics of dental performance. The simulator delivers a highly realistic pictorial representation of the presented stimuli (e.g. the oral cavity and teeth) with high face validity^29^ and good construct validity.^30^ The digital nature of the simulator then allowed a highly ecological examination of the difference in performance when stereoscopic cues were present and when they were absent (with a rich monocular field of view available in the absence of stereo‐information).

The participants used a physical hand piece and a dental mirror both with a virtual tip that appears on the simulator screen, to perform tooth preparation procedures with realistic sound rendering. The speed of the virtual hand piece is controlled using a real foot pedal. In order to mimic the reality of the real‐world task, participants were able to freely move their head. This meant that in principle they could obtain depth information via motion parallax – but at the cost of the postural instability induced by the biomechanics of head movements.

For this study, we used the manual dexterity exercises developed by ACTA (Academic Center for Dentistry, Amsterdam, The Netherlands; www.acta.nl) used previously to examine motor skill.^31^ The simulator outputs stereo information via two digital multimedia projectors, which operate simultaneously, resulting in the projection of two images superimposed onto the screen through a polarising filter. The screen size is 5″ with a refresh rate of 60 Hz and a resolution of 800 × 600. Importantly, the screen size is ‘life‐sized’ and so accurately represents the physical hand piece, which is ‘mirrored’ in the co‐located visual display. The users wear passive polarised glasses to perceive the 3D image.^23^ The simulator was engineered to output a single image (to both eyes) within the bi‐ocular condition.

### Experimental tasks

**Figure 1:**
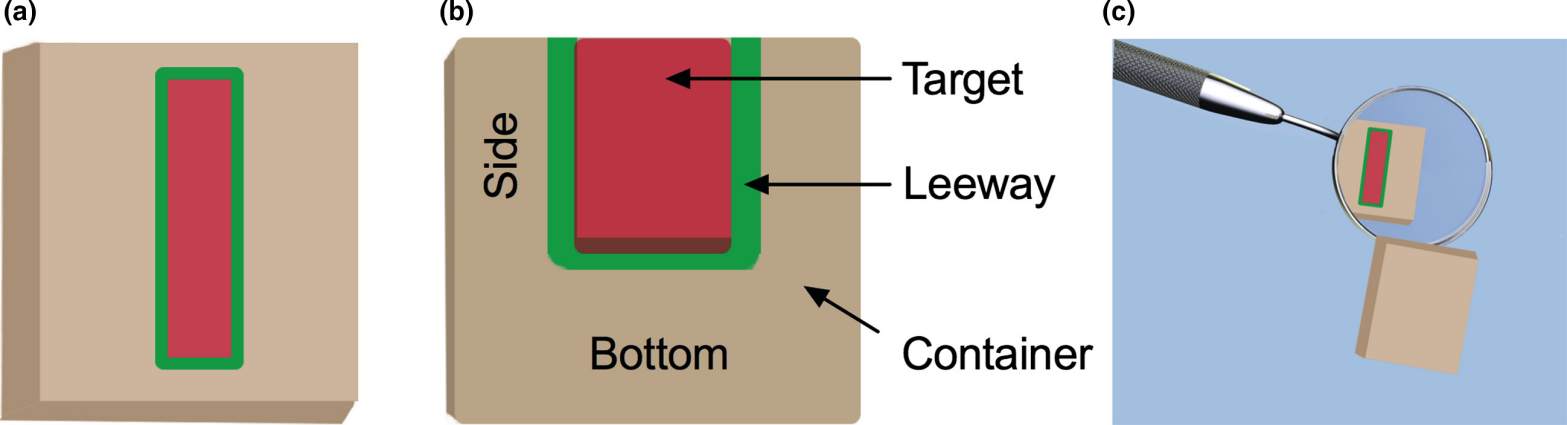
(a) Top‐view of the abstract shapes used for the drilling task. (b) Side view with cross‐section shows the different regions of the shape. Drilling in the sides and bottom of these regions comprised the error metrics for laterality and depth, respectively. (c) Each drilling task was completed via indirect (with the shape reflected through a mirror‐ as pictured here) and direct (no mirror) observation. [Colour figure can be viewed at wileyonlinelibrary.com]

Each participant performed four different tasks from the manual dexterity module in Simodont. The tasks differed in target shape with two basic abstract shapes with minimal geometric difference chosen (*Figure* 1a). The rationale of choosing the two slightly different shapes was to ensure that the performance was not due to practice effect (i.e. performing the same shape four times) and at the same time to ensure that both shapes were not markedly different in geometry, which may add a difficulty factor that is not intended in the current experiment. The different regions of the abstract shape are illustrated in cross section (*Figure* 1b).

Participants performed each task with two viewing orientations: directly viewing the shape and indirectly viewing via a virtual dental mirror (*Figure* 1c). Participants were instructed to drill/cut as much of the target region (red) as possible whilst minimising drilling in the leeway (green) and container (beige) regions. There were no haptic cues regarding the region of operation so participants needed to use visual information to control their performance (the simulator provided full haptic feedback but there were no changes in the haptic feedback if the participants left the target area). The target area was 1.5–2 mm wide with depths of 1.5 mm, 0.8 mm or 0.4 mm. The beige container was 10 mm long by 10 mm wide by 2 mm deep and the green leeway space surrounding the bottom and side of the target area was 0.2 mm. The instructions to the participants emphasised drilling accuracy and reaching the acceptable target removal percentage (60%). Participants were instructed to drill/cut as much of the target region as possible whilst minimising drilling in the leeway and container regions (it is worth reiterating that these were experienced dentists). There were a number of cues to indicate target depth even without a stereoscopic view (as is the case in real‐world dentistry). The target area was red in color so the participant could see this color until it was removed (in the same way that caries provides color cues in real teeth). Moreover, whenever the participant drilled there was a visible indentation (deformation) in the shape. It was therefore possible for the participants to perform the task without the additional information conveyed by a stereoscopic view.

Once the participant pressed the foot pedal the hand piece with its attached cutting tool (dental bur) started revolving. Once the bur came in contact with the block the drilling/cutting took place providing that the participant pressed on the specific area. Meanwhile, the computer screen attached to the simulator showed all the recorded metrics in details (e.g. the percentage of the target removed, the percentage of errors to the sides and bottom of the shape). Therefore, the participant was able to monitor his/her progress in real time.

The participants were able to freely move (rotate and tilt) the test block (abstract shape) with the control handle located beneath the display screen, so that it was possible for them to access the target area conveniently (as it is possible in a real clinical case where the patient position can be adjusted to access the tooth being treated). To avoid any confounding order effects, we counterbalanced the task shape order as well as the task viewing orientation among participants. The recorded results related to one trial per participant.

### Data collection and statistical analysis

We used a cross sectional quantitative study with a repeated measure design. Dental task performance was captured using the metrics provided automatically by the simulator: error scores (Leeway and Container) for the sides and the bottom of the abstract shape, and the task completion percentages. The Simodont creates a volume of data, much like a CT scan, where the space is divided into small ‘cubes’ (voxels) of length 200 microns. Each voxel is labelled according to the zone it represents (Target, Leeway Sides, Leeway Bottom etc.). As the operator drilled the template, the proportion of voxels removed from each zone was recorded automatically. Error scores were created based on the proportion of each zone removed. For this study, we were primarily concerned with the amount of depth related drilling error exhibited by participants within each condition. To this end, we used a composite score of the amount of drilling error in the Leeway bottom and Container Bottom regions of the abstract shape (quantified as the amount of area drilled as a percentage of total surface area) to identify Depth Error (DE). Given that errors in the container should be considered to be of a greater magnitude than those in the leeway range, we also examined these metrics separately. Only a small proportion of the drilling for the population sampled (qualified dentists) was in the container area (mean depth errors = 0.56%, mean lateral errors = 0.31%) and as such, the leeway measure showed the same pattern of results as the composite measure. Thus, for brevity, we report only the composite measure results here and report analyses on individual metrics in Data S1.

We also calculated the total error scores made by the participant at two other specific areas of the abstract shape (the leeway and container sides) to produce a composite score for Lateral Error (LE). Finally, the percentage of total surface area removed from the Target region of the shape provided was used to measure Target Area Removal.

Each dependent variable was subjected to a 2 (Vision Type [Bi‐ocular vs Binocular] X 2 (Orientation [Direct vs Indirect]) repeated measures ANOVA. All data were tested for departures from normality by boxplot, Q‐Q plots, histograms and a Shapiro – Wilk test, with transformations performed where necessary. The statistical significance threshold was set to *p *<* *0.05. Bonferroni‐corrected post hoc comparisons were performed where significant main effects were found. Partial eta squared values ($\eta_{p}^{2}$) are reported to indicate effect size. All statistical analyses were performed using IBM SPSS^®^ Statistics for Windows (Version 22, Armonk, NY, USA: IBM Corp., 2013).

## Results

### Depth

**Figure 2:**
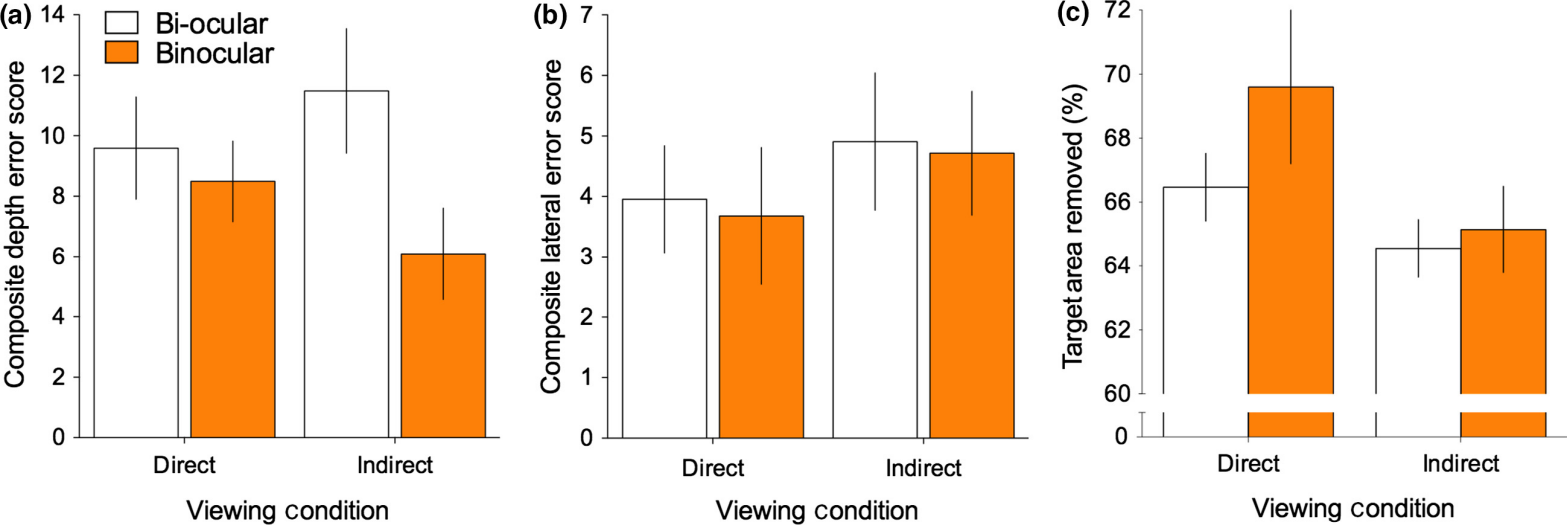
Drilling task performance in Bi‐ocular vs Binocular vision under direct and indirect viewing orientation for (a) depth‐related errors, (b) lateral errors and (c) target area removal. Error bars represent ± 1 S.E.M. [Colour figure can be viewed at wileyonlinelibrary.com]

**Figure 3:**
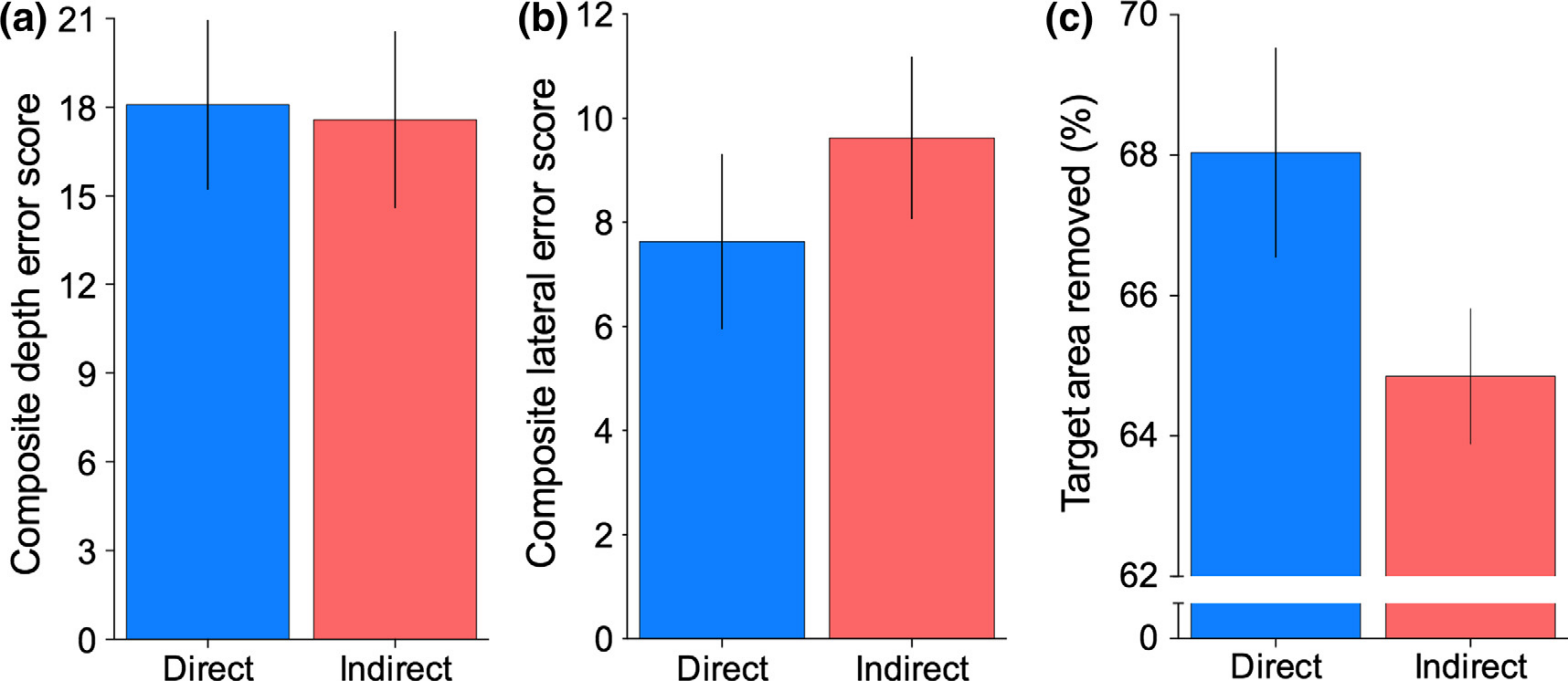
Drilling task performance measures under Direct and Indirect viewing orientation for (a) depth errors, (b) lateral errors and (c) Target area removal. Error bars represent ± 1 S.E.M. [Colour figure can be viewed at wileyonlinelibrary.com]

The impact of the type of visual information available to perform the tasks (Bi‐ocular vs. Binocular) on depth errors was statistically significant, *F*_1,12_ = 7.55, *p *=* *0.02, $\eta_{p}^{2}$ = 0.38, with mean drilling error being higher for the Bi‐ocular viewing condition (*M *=* *21.10, S.E.* *= 2.96) compared to Binocular (*M *=* *14.62, S.E. = 2.41; *Figure* 2a). These results indicate that participants were drilling too far in the Bi‐ocular viewing condition. Interestingly, the effect of task orientation on depth errors (*Figure* 3a) was not statistically significant, *F*_1,12_ = 0.02, *p *=* *0.88, $\eta_{p}^{2}$ = 0.002, nor was the interaction between the vision manipulations and orientation, *F*_1,12_ = 1.90, *p *=* *0.19, $\eta_{p}^{2}$ = 0.14.

### Lateral errors

In contrast to drilling depth errors, the type of visual information available to perform the tasks had no statistically significant effect on the amount of lateral errors made by participants (*Figure* 2b), *F*_1,12_ = 0.06, *p *=* *0.81, $\eta_{p}^{2}$ = 0.01. In addition, there was no effect of orientation (Direct vs Indirect; *Figure* 3b), *F*_1,12_ = 0.93, *p *=* *0.35, $\eta_{p}^{2}$ = 0.07 and no significant interaction between vision and orientation the amount of lateral drilling error, *F*_1,12_ = 0.002, *p *=* *0.96, $\eta_{p}^{2}$ < 0.01.

### Target area removal

The effect of the type of vision (Bi‐ocular vs. Binocular) did not statistically influence the total amount of target area removal (*Figure* 2c), *F*_1,12_ = 2.74, *p *=* *0.13, $\eta_{p}^{2}$ = 0.18. However, reflecting the increased difficulties associated using indirect (mirror) observations, we did find a significant main effect of orientation (*Figure* 3c). *F*_1,12_ = 4.81, *p *=* *0.05, $\eta^{2}$ **= 0.28, with higher target area removal scores for Direct observation (*M *=* *68.04, S.E. = 1.49) compared to Indirect (*M *=* *64.91, S.E. = 0.96). The interaction between vision type and orientation on target area removal did not reach statistical significance, *F*_1,12_ = 0.82, *p *=* *0.38, $\eta_{p}^{2}$ = 0.06.

The mean values for all performance metrics under the experimental conditions are presented in (*Table* 1).

**Table 1:** Mean (± S.D.) for the performance metrics recorded in the current study under the experimental condition (*N* = 13)

		Vision			
		Non‐stereoscopic		Stereoscopic	
		Task viewing orientation		Task viewing orientation	
		Direct	Indirect	Direct	Indirect
Performance metrics	Depth errors composite (%)	9.6 (±6.1)	11.5 (±7.4)	8.5 (±4.8)	6.1 (±5.5)
	Lateral errors composite (%)	3.9 (±3.2)	4.9 (±4.1)	3.6 (±4.1)	4.7 (±3.6)
	Target area removal (%)	66.5 (±3.8)	64.5 (±3.3)	69.6 (±8.6)	65.2 (±4.8)

## Discussion

We investigated the role of stereopsis in performance on a basic simulated dental task with qualified dentists. The data clearly showed that the participating dentists benefitted from the presence of stereovision within the current experimental setting. Importantly, we demonstrated that the presence of stereovision decreased depth‐related drilling errors but not lateral errors. These findings make sense as the monocular pictorial cues provide unambiguous information on the impact of drilling on the lateral extent of the tooth. In contrast, there are few direct cues to depth available from a monocular pictorial representation and this explains why stereopsis is so important for skilled performance. The pattern of results allows confidence that the experimental manipulation was specific to the hypothesised role of stereopsis (i.e. improving depth perception) rather than a general decrement in performance induced by unusual viewing conditions.

We also found a reliable effect of observation, with lower target area removal in the indirect (mirror) condition made when compared to direct observation. This is not surprising because indirect tasks (performed with mirror vision) are inherently more challenging and impose additional task challenges in terms of hand‐eye coordination, dental mirror positioning and hand piece control. It may also be that other kinematic measures (such as the smoothness of drilling – which can be obtained from the mathematical derivative of jerk – would capture the performance differences between indirect and direct observation. The other factor that might have been affected is the duration of the dental procedure. In the present experiments, the participants were experienced dentists (at least 3 years of clinical experience) and were not placed under artificial time constraints (and all participants completed the procedures within the normal expected duration for such tasks). It has been shown previously (with reach‐to‐grasp behaviour) that the removal of binocular vision results in slower (more cautious) movements 8, 32 that can affect task completion times, especially for more difficult activities, such as bead threading.^11^ It is possible, therefore, that the impact of removing stereoscopic information is even higher when dentists are operating in time constrained situations ‐ though this is a conjecture that needs empirical investigation.

We were interested to see whether there was an interaction between the viewing conditions and whether observation was direct or indirect. In fact, we found no statistically reliable interaction on any of our measures. It is always difficult to interpret a null finding and the lack of an interaction may simply reflect a lack of statistical power. The results do, however, suggest that the largest effects on performance are driven by the presence or absence of stereo information and the task complexity. It is worth emphasising again that these findings relate to experienced dentists and a different pattern of results might be found in novices. It seems reasonable to assume that stereovision will play a larger role in the more difficult condition of indirect viewing for novices (as the usefulness of this information is likely to be higher when the skill is still be mastered). More generally, stereovision may play a larger role during the early stages of dental skill acquisition (before the novices become tuned to the monocular pictorial cues) though this is a conjecture that, again, needs empirical investigation. We note, however, that there is existing evidence suggesting stereovision plays an important role in the learning related to one‐handed ball‐ catching.^33^, ^34^ This provides a more general context to our specific conjecture that stereopsis may play an important role in the acquisition of dental surgery skills.

The participants within this study had normal stereoacuity and presumably refined their dental skills on the basis of this information being available. It is possible that individuals with long‐term stereo‐deficits may learn to use other sources of information (e.g. knowledge of dental anatomy, motion parallax cues or the use of a gauging instrument such as a periodontal probe) and thereby avoid a reliance on stereopsis. The possibility of other cues being used has been explored within previous research exploring the role of stereopsis in reaching‐to‐grasp,^35^ where it was found that individuals with permanent stereo‐deficits show performance decrements (i.e. these individuals have not been able to compensate for their stereo‐deficits). There is a need, however, to determine specifically whether it is possible to compensate for long‐term stereo‐deficits in dental skills. This will require the identification of qualified dentists with stereo‐deficits and comparing their performance with dentists who have normal stereoacuity. We argue that such research is urgently needed so that the needs of patients can be balanced with appropriate selection criteria that do not unfairly discriminate against individuals who have a deficit but which has no functional significance within the occupation.

The present findings also have implications for the design of dental simulators. There has been an assumption that stereovision is an important feature of such simulators. The data presented within this paper provide empirical support for this assumption. Unfortunately, however, the simulators do not track the head position of the simulator user. This is problematic as horizontal retinal image disparities are a function of viewing distance and angle. Furthermore, the types of monocular cues that are typically available in natural environments that might potentially compensate for stereopsis (e.g. shadows caused by the operator) are not fully simulated whilst the presence of ghosting (crosstalk) on the projection screen could also impact on performance‐ an issue that needs careful consideration in simulator design more broadly.^36^, ^37^ The fact that dentists are learning to use stereopsis to control their actions suggests that simulators should ensure the perceptual information used in training maps to the information available in the real world. We therefore argue that simulators should track head position and control for inter‐pupillary distance to ensure that disparities are rendered accurately within the displays.

The present study focussed on the use of stereopsis in dentistry. It is worth noting, however, that the results have implications for other surgical disciplines – most notably ophthalmology where surgeons need to control precisely the position of instruments in depth. The current results lead to the direct prediction that ophthalmologists will benefit from stereoscopic information. The data also suggest that ophthalmology simulators should include a veridical stereoscopic view. There are further implications for clinical eye services ‐ it has hitherto been difficult for an ophthalmologist, optometrist or orthoptist to advise a patient on the implications of a detected stereo‐deficit. The present results suggest that ignoring a stereo‐deficit in a child may impact on their professional opportunities at a later stage in their life. The results also raise issues for professions that require a certain level of perceptual‐motor skill (including, ironically, some areas of ophthalmology and optometry). These professions might need to restrict training opportunities to individuals with a minimum level of stereoacuity on health and safety grounds. However, the lack of reliable data on the significance of stereopsis means that professions need to either err on the side of admitting individuals who might lack the requisite perceptual abilities or risk discriminating against individuals who have a deficit that has no functional significance.

Finally, we note that dental tasks vary widely in terms of their complexity and this is likely to affect the need for stereoscopic information for successful performance. In the current study, the tasks were basic manual dexterity exercises that were controlled in terms of the standardised settings within which the dental cutting (removing the target area) is performed. The ‘teeth’ were positioned evenly in the participant's view and were presented in a uniform virtual block with no adjoining dental structures. It seems reasonable to assume that the role of stereopsis will increase as the perceptual‐motor demands of the task increase.^2^, ^11^, ^15^ The fact that we found an effect of removing stereopsis in relatively simple tasks indicates the fundamental role for stereopsis in eye‐hand coordination. More specifically, this work empirically demonstrates the functional significance of stereopsis within a professional setting. There is a need to determine more widely what occupations likewise require stereopsis to ensure the health and safety of the individuals who rely on the skills of the professionals within that discipline.

## Disclosure

The authors report no conflicts of interest and have no proprietary interest in any of the materials mentioned in this article.

## Supporting information


**Data S1.** Statistical analyses on depth and lateral errors for each region of the workspace.
