# Supplementary material for: Drilling into the functional significance of stereopsis: the impact of stereoscopic information on surgical performance
Source: Ophthalmic Physiol Opt. 2017 Jun 27;37(4):498–506. doi: 10.1111/opo.12393 (PMC5519940; doi:10.1111/opo.12393)
Supplement: Supplementary file 1 — Data S1. Statistical analyses on depth and lateral errors for each region of the workspace. [file OPO-37-498-s001.pdf]

Supplementary Information

The main paper describes results from a composite measure combining errors made in the Leeway and Container regions of the task environment. Here, we report analyses on depth and lateral errors for each region.

Leeway Region

Consistent with the composite measure analysis, the influence of the type of visual information available to perform the tasks (Bi-ocular vs. Binocular) was statistically significant for Leeway depth-related errors, F (1,12) = 8.46, p = .013, η_p^2= .41 (see Figure S1A). The effect of viewing orientation was not significant, F (1,12) =0.124, p=.730, η_p^2= .01, and the interaction between the vision manipulations and orientation was also not statistically significant, F (1,12) = 3.17, p = .10, η_p^2 = .21.

For lateral errors, we found no statistically reliable difference for Vision Type, F (1,12) = .06, p = .81, η_p^2< .01. The effect of task orientation on Leeway lateral errors was also not significant, F (1,12) =0.59, p= .46, η_p^2=0.05, nor was the interaction between the vision manipulations and orientation, F (1,12)= .2, p = .66, η_p^2 = .02. The mean Leeway lateral errors are shown in Figure S1B.

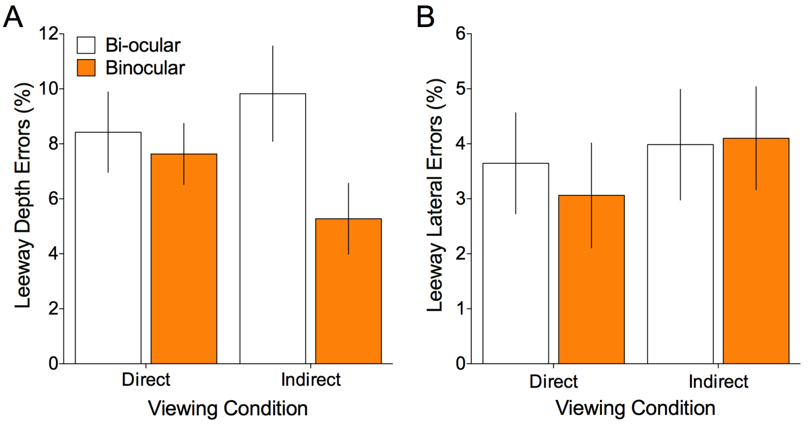

Figure S1 Drilling task performance for Bi-ocular vs. Binocular vision under Direct and Indirect viewing orientations for (A) depth-related errors (B) lateral errors in the Leeway region. Error bars represent ± 1 S.E.M.

Container Region

Given that the sample we tested were qualified dentists, only minimal amounts of drilling was observed in this region. Furthermore, assumptions of normality for container error scores were violated as assessed by Shapiro-Wilk's test ($p <. 05$). Transformations did not yield normally distributed values, therefore, a non-parametric test (Wilcoxon signed-rank test) was used to analyze performance differences for Vision Type (Bi-ocular vs. Binocular) and Orientation (Direct vs Indirect) separately and these results are reported below.

The effect of the type of visual information available to perform the tasks on container depth errors did not the reach statistical significance threshold, as assessed Wilcoxon signed-rank test $z = -1.69$, $p = .09$. There was also no effect of orientation on container depth errors ($z = .54$, $p = .59$). The mean container depth errors are shown in Figure S2. For lateral errors, there was no effect of Vision Type (Wilcoxon signed-rank test $z < .01$, $p = 1$) and no effect of Task Orientation ($z = 1.27$, $p = .21$).

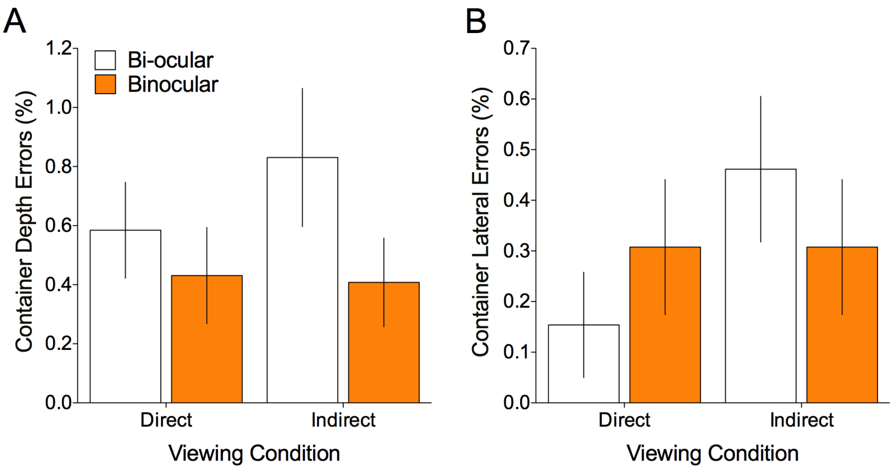

Figure S2 Drilling task performance for Bi-ocular vs. Binocular vision under Direct and Indirect viewing orientations for (A) depth-related errors and (B) lateral errors in the Container region. Error bars represent ± 1 S.E.M.
